# Allopolyploid speciation and ongoing backcrossing between diploid progenitor and tetraploid progeny lineages in the *Achillea millefolium *species complex: analyses of single-copy nuclear genes and genomic AFLP

**DOI:** 10.1186/1471-2148-10-100

**Published:** 2010-04-13

**Authors:** Jin-Xiu Ma, Yan-Nan Li, Claus Vogl, Friedrich Ehrendorfer, Yan-Ping Guo

**Affiliations:** 1 Ministry of Education Key Laboratory for Biodiversity Science and Ecological Engineering, and College of Life Sciences, Beijing Normal University, Beijing 100875, China; 2 College of Biological Sciences and Biotechnology, Beijing Forestry University, Beijing, China; 3 Institute of Animal Breeding and Genetics, University of Veterinary Medicine in Vienna, A-1210 Vienna, Austria; 4 Department of Systematic and Evolutionary Botany, Faculty of Life Sciences, University of Vienna, A-1030 Vienna, Rennweg 14, Austria; 5 Beijing Engineering Research Center for Hybrid Wheat, Beijing 100097, China

## Abstract

**Background:**

In the flowering plants, many polyploid species complexes display evolutionary radiation. This could be facilitated by gene flow between otherwise separate evolutionary lineages in contact zones. *Achillea collina *is a widespread tetraploid species within the *Achillea millefolium *polyploid complex (Asteraceae-Anthemideae). It is morphologically intermediate between the relic diploids, *A. setacea*-2x in xeric and *A. asplenifolia*-2x in humid habitats, and often grows in close contact with either of them. By analyzing DNA sequences of two single-copy nuclear genes and the genomic AFLP data, we assess the allopolyploid origin of *A. collina*-4x from ancestors corresponding to *A. setacea*-2x and *A. asplenifolia*-2x, and the ongoing backcross introgression between these diploid progenitor and tetraploid progeny lineages.

**Results:**

In both the ncp*GS *and the *PgiC *gene tree, haplotype sequences of the diploid *A. setacea*-2x and *A. asplenifolia*-2x group into two clades corresponding to the two species, though lineage sorting seems incomplete for the *PgiC *gene. In contrast, *A. collina*-4x and its suspected backcross plants show homeologous gene copies: sequences from the same tetraploid individual plant are placed in both diploid clades. Semi-congruent splits of an AFLP Neighbor Net link not only *A. collina*-4x to both diploid species, but some 4x individuals in a polymorphic population with mixed ploidy levels to *A. setacea*-2x on one hand and to *A. collina*-4x on the other, indicating allopolyploid speciation as well as hybridization across ploidal levels.

**Conclusions:**

The findings of this study clearly demonstrate the hybrid origin of *Achillea collina*-4x, the ongoing backcrossing between the diploid progenitor and their tetraploid progeny lineages. Such repeated hybridizations are likely the cause of the great genetic and phenotypic variation and ecological differentiation of the polyploid taxa in *Achillea millefolium *agg.

## Background

According to the genealogical species concept, species are defined as multi-locus "genotypic clusters" that remain distinct even in the presence of gene flow among each other [1-3]. "Hybridization is thus a normal feature of species biology" [1]. Hybridization and its results, e.g., introgression, segregation of new types without backcrossing, and allopolyploidy, have long been speculated as major forces behind "evolutionary bursts" [4]. Indeed, plant species and populations arisen from hybridization and polyploidy often exhibit more complicated patterns of variation than their progenitors, i.e., their diploid sister groups, and are ecologically divergent, presumably under local selection. Furthermore, when gene flow is present between the diverged progenies or between the parental and daughter lineages, the genetic and phenotypic complexity of the populations could be enhanced. All these processes may increase species diversity and obliterate discrete separation lines between otherwise diverged taxa as observed in many angiosperm polyploid complexes [4-9].

*Achillea millefolium *agg. (Asteraceae-Anthemideae) is a highly polymorphic but clearly monophyletic polyploid species complex or aggregate. It is composed of outbreeding hemicryptophytic perennials widely distributed over the N Hemisphere. Five to seven diploid and 10-30 polyploid taxa can be defined in this complex [10,11]. Autopolyploidy has been documented in the N American populations, which serve as textbook examples for plant ecotypic differentiation [12,13]. Most of the Eurasian polyploids, ranging from tetra- to octoploids, are either derived from primary hybridization between diploid progenitors or may be products of secondary introgression on the same or on different ploidy levels. This has created complex genetic and phenotypic variation patterns within *A. millefolium *agg. [14-18]. The relationships of the diploid species conform to a tree structure, whereas most of the polyploid taxa exhibit complex and reticulate relationships with each other and with the diploid species [11,19].

*Achillea collina *is a widely distributed tetraploid member of *A. millefolium *agg. in Europe. It is morphologically intermediate between the relic diploids, *A. setacea*-2x in xeric and *A. asplenifolia*-2x in humid habitats, and often grows in close contact with either of them [14]. Cytogenetic analyses and crossing experiments of *A. asplenifolia *and *A. setacea *have resulted in F_1 _and F_2 _generations with reduced vitality and fertility. Thus, the two diploid species are separated by considerable intrinsic barriers. From their diploid F_2 _hybrid progeny, several spontaneous allotetraploid individuals could be obtained. They were morphologically quite similar to the wild species *A. collina*-4x, fertile, and could be crossed with the latter [14]. Previous AFLP analyses have suggested *A. setacea*-2x and *A. asplenifolia*-2x as the most likely progenitors of *A. collina*-4x [19]. In the Austrian province of Burgenland, south of Vienna, we found several natural hybrid swarms where either morphologically "typical" *A setacea*-2x or "typical" *A. asplenifolia*-2x come into contact with *A. collina*-4x. We suspect some 4x plants in these hybrid zones to be products of backcrosses from *A. setacea*-2x or *A. asplenifolia*-2x via unreduced egg cells to their assumed daughter species *A. collina*-4x. Clarification of genetic relationships of these diploid and tetraploid individuals and populations should improve our understanding of the enormous species diversity and the complex patterns of variation in *A. millefolium *agg..

To resolve reticulate relationships and recent radiation, single- or low-copy nuclear genes are preferable because *i*) they can provide co-dominant molecular markers for identifying hybridization and/or introgressive events, *ii*) they often provide multiple unlinked loci with fast evolving introns, and are thus more informative than the plastid DNA, *iii*) such low-copy nuclear loci are less susceptible than ribosomal genes to gene conversion, which can reduce or eliminate allelic heterozygosity. The major problem in utilizing low-copy nuclear genes is to distinguish orthologs from paralogs. Only with orthologs, phylogenetic interpretations make sense [20-22]. In addition, PCR-recombination can also be a problem when sequencing nuclear genes, especially from polyploid genomes. When two partially homologous templates exist in one PCR reaction, an in vitro chimera could be formed from the non-identical templates. This can happen when amplifying members of multigene families or any locus from polyploid genomes [23,24]. By optimizing PCR conditions, the frequency of PCR recombination can be reduced [24]. Nevertheless, data should be interpreted cautiously to avoid biased evolutionary interpretations due to artificially recombinant molecules [23].

With large numbers of markers, the AFLP method can help to obtain genome-wide perspectives about populations under processes influencing the entire genome, such as gene flow and genetic drift. Therefore, this is a powerful tool in recognizing hybridization events [19,25,26].

Here we use sequences of two single-copy nuclear genes, the chloroplast-expressed glutamine synthase gene (ncp*GS*) and the cytosolic phosphoglucose isomerase gene (*PgiC*) as well as AFLP data to demonstrate allopolyploid speciation and ongoing hybrid introgression by backcrossing between diploid progenitor and tetraploid progeny lineages in *Achillea millefolium *agg..

## Results

### Genealogical relationships based on the nuclear gene sequences

**Figure 1:**
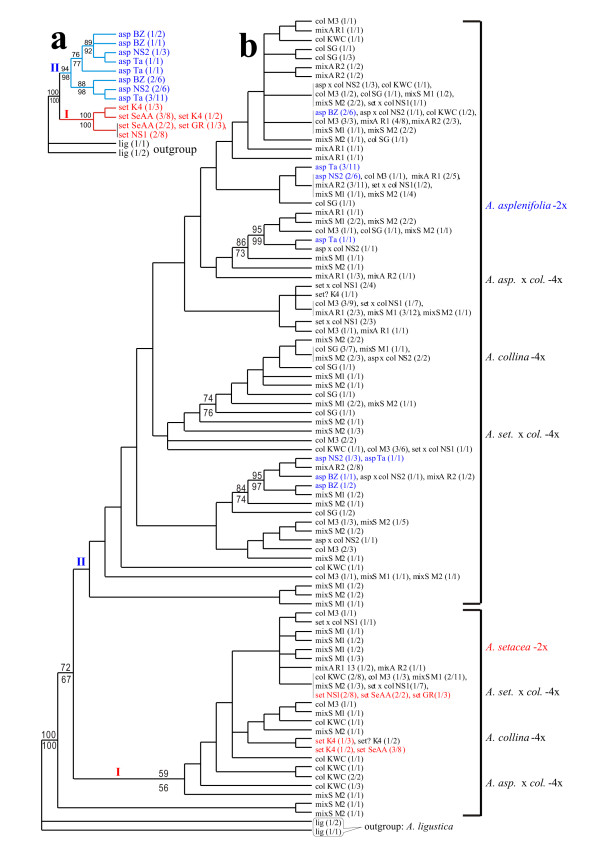
**Maximum parsimonious (50% majority-rule consensus) trees of the ncp*GS *gene. a**. For the diploid species *Achillea setacea *and *A. asplenifolia *only based on 13 consensus sequences and two equally most parsimonious trees (tree length = 119, CI = 0.8824, RI = 0.9343). **b**. For all the studied diploid and tetraploid species and populations based on 80 consensus sequences and 8700 equally most parsimonious trees (tree length = 403, CI = 0.4491, RI = 0.8649). Bootstrap supports (>50%) from MP/NJ analyse are shown above/below the major branches. **Label** for the sequence (terminal node) is written as "taxa abbreviation # population code (number of individuals/number of clones)". **Abbreviations**: asp = *A. asplenifolia*-2x, set = *A. setacea*-2x, col = *A. collina*-4x, mixA = mixed populations of *A. asplenifolia*-2x and suspected *A. asplenifolia *x *collina*-4x backcross individuals, mixS = mixed populations of *A. setacea*-2x and suspected *A. setacea *× *collina*-4x backcross individuals.

**Figure 2:**
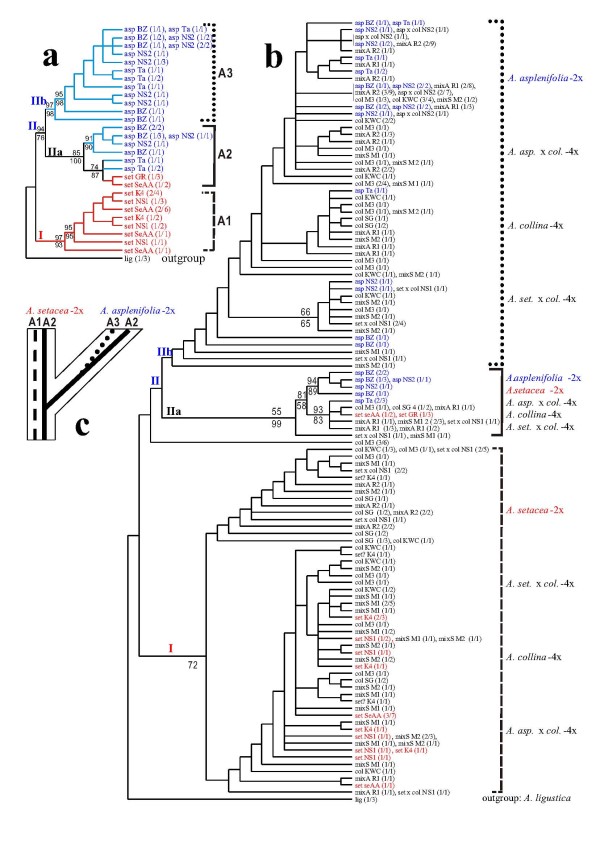
**Maximum parsimonious (50% majority-rule consensus) trees of the *PgiC *gene**. **a**. For the diploid species *Achillea setacea *and *A. asplenifolia *only based on 29 consensus sequences and 14 equally most parsimonious trees (tree length = 212, CI = 0.6415, RI = 0.9007). **b**. For all the studied diploid and tetraploid species and populations based on 109 consensus sequences and 7840 equally most parsimonious trees (tree length = 508, CI = 0.3484, RI = 0.8909). Bootstrap supports (>50%) from MP/NJ analyse are shown above/below the major branches. **Label **for the sequence (terminal node) is written as "taxa abbreviation # population code (number of individuals/number of clones)". **Abbreviations**: asp = *A. asplenifolia*-2x, set = *A. setacea*-2x, col = *A. collina*-4x, mixA = mixed populations of *A. asplenifolia*-2x and suspected *A. asplenifolia *× *collina*-4x backcross individuals, mixS = mixed populations of *A. setacea*-2x and suspected *A. setacea *× *collina*-4x backcross individuals. **c**. Proposed scheme of incomplete lineage sorting of the *PgiC *gene. Species are outlined by thin solid lines; alleles A1, A2 and A3 are represented by dashed, thick solid, and dotted lines, respectively.

Amplifications for both the ncp*GS *and the *PgiC *locus yielded a single band from each individual sample. The ncp*GS *haplotype sequences of the 2x individuals and populations group into two clades corresponding to the two diploid species (Fig. 1a), thus clearly belong to a set of single-copy orthologs. The *PgiC *gene tree does not completely correspond to the divergence of the diploid species (Fig. 2a). This can be attributed to incomplete sorting of two ancestral *PgiC *alleles in *Achillea millefolium *agg. (Fig. 2c) or to introgression (for detailed interpretation, see the "Discussion"). Therefore, all the *PgiC *sequences studied here also belong to one orthologous gene lineage.

The original complete ncp*GS *data matrix contains 327 sequences (clones) from 60 individuals of 14 studied populations and the outgroup *A. ligustica*. The final ncp*GS *gene tree (Fig. 1b) was built on 80 consensus sequences ranging in length from 873 to 921 bps. The alignment contains 971 nucleotide positions, of which 869 (195 in exon and 666 in intron regions) were included in the phylogenetic analysis containing 151 parsimony-informative characters.

The original complete *PgiC *data set contains 252 sequences (clones) from the same 59 out of 60 individuals analyzed for the ncp*GS *locus. The final *PgiC *gene tree (Fig. 2b) was built on 109 consensus sequences ranging in length from 1619 to 1674 bps. The alignment contains 1720 nucleotide positions, of which 1579 (646 in exon and 933 in intron regions) were included in the phylogenetic analysis containing 127 parsimony-informative characters.

A heuristic search retained 8700 equally most parsimonious (MP) trees (tree length = 403, CI = 0.4491, RI = 0.8649) from the 80 consensus ncp*GS *sequences and 7840 MP trees (tree length = 508, CI = 0.3484, RI = 0.8909) from the 109 consensus *PgiC *sequences. Topologies of the MP and NJ trees were broadly similar. Figs. 1 and 2 show the 50% majority rule consensus MP trees. Internal node supports (Bootstrap Percentages) from both MP and NJ methods were presented on the trees.

Phylogenetic analyses were first conducted for the diploid species only (Figs. 1a &2a). Rooted by the Central Mediterranean *Achillea ligustica*-2x, each of the gene trees contains two well supported clades: clade **I **corresponds to *A. setacea*-2x in both gene trees, and clade **II **in the ncp*GS *tree to *A. asplenifolia*-2x only, whereas in the *PgiC *tree, subclade **IIa **(haplotype group A2) contains sequences not only of *A. asplenifolia*-2x, but also a few of *A. setacea*-2x (populations SeAA and GS from Anatolia and Greece). We interpret the haplotype group **A2 **orthologous to **A1 **and **A3**, and designate A1 and A2 as polymorphic alleles of the *PgiC *gene from the ancestral lineage of *A. millefolium *agg. (more in the "Discussion" part).

In contrast to the diploid individuals and populations, the tetraploid *A. collina *and its suspected backcross hybrids in the polymorphic "mixed" populations show homeologous copies at both ncp*GS *and *PgiC *loci. In most cases, different sequences from the same tetraploid individual plant were placed in different diploid clades (Figs. 1b &2b; Additional files 1 and 2: Figs. S1 & S2).

### AFLP split network

**Figure 3:**
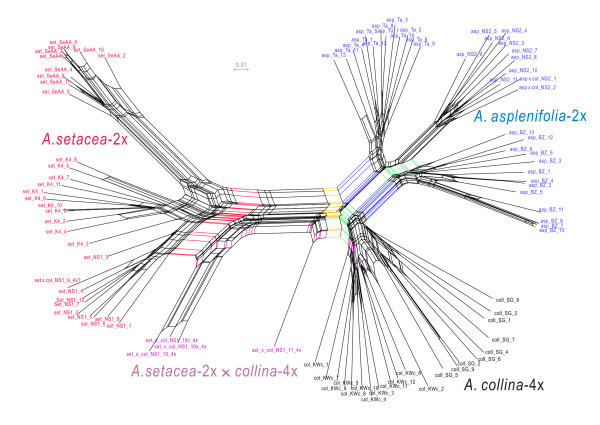
**Neighbor Net derived from 273 AFLP bands of 93 individuals from eight populations of the two diploid progenitor lineages *Achillea setacea-*2x and *A. asplenifolia*-2x, the allotetraploid *A. collina*-4x and backcross individuals**. Node labels include taxa abbreviations (asp = *A. asplenifolia*-2x, set = *A. setacea*-2x, col = *A. collina*-4x) and population codes.

Three primer pairs generated a total of 273 clear and unambiguous AFLP bands from 93 individuals of eight populations. Out of the 273 bands, 245 (89.7%) were polymorphic. The 4x-accessions have more bands (average 127.1 bands per individual) than the 2x ones (average 115.6 bands per individual in *A. asplenifolia*-2x and 114.2 in *A. setacea*-2x). Thirty-seven differences of 4386 phenotypic comparisons were observed based on the 17 replicated individuals, thus the error rate is 0.84%. Fig. 3 shows a Neighbor Net of the 93 individuals studied by the AFLP method. Two major splits, highlighted by red and blue, correspond to *A. setacea-*2x and *A. asplenifolia*-2x, respectively. The box formed by the semi-congruent blue and green splits indicates the hybrid status of *A. collina*-4x. The incompatible yellow and purple splits link the *A. setacea *× *collina-*4x individuals from population NS1 to *A. setacea *on the one hand and to *A. collina *on the other, demonstrating backcross introgression between the latter two.

## Discussion

*Achillea setacea *and *A. asplenifolia *are two diploid species of the monophyletic *A. millefolium *agg. [11,19]. They represent two extremes of morphological and ecological differentiation within this species aggregate, the former hairy, small, and adapted to xeric steppe environments, the latter tall, glabrous, and adapted to undisturbed wet environments. *Achillea setacea*-2x is sporadically distributed from NE Anatolia and SE Europe to the Balkans, Hungary, Slovakia, Moravia, Austria and interior valleys of the Alps, and in the north to S Poland, E Germany and the N Czech Rep; whereas, *A. asplenifolia*-2x occurs locally from Bulgaria and Hungary to E Austria and the southern Czech Republic [10,11,27]. In the ncp*GS *gene tree, haplotype sequences of *A. setacea*-2x and *A. asplenifolia*-2x group well into two clades corresponding to the two species (Fig. 1a), the *PgiC *gene tree, however, does not completely correspond to the divergence of the diploid species (the subclade IIa of Fig. 2a inclues both *A. asplenifolia*-2x and *A. setacea*-2x) (Fig. 2a). Our data clearly show that both the ncp*GS *and *PgiC *genes are single-copy in *Achillea millefolium *agg.. To explain the partial incongruence of the *PgiC *gene tree with the divergence of the diploid species (Fig. 2a), two interpretations can be put forward: *i*) incomplete sorting of ancestrally polymorphic alleles, or *ii*) of introgression during secondary contact of the two diploid species. Considering the current allelic distribution, the former interpretation is more likely as shown below.

Assuming incomplete lineage sorting (Fig. 2c) [28], allele **A2 **might have been retained from an ancestor of *A. millefolium *agg. in some populations of the extant *A. setacea *(the Greek and Anatolia populations, GR and SeAA) and in *A. asplenifolia*, but was apparently lost during the migration of *A. setacea *to the north and the west, e.g., in the Ukrainean and Austrian populations (K4 and NS1). Allele **A3**, which appears in *A. asplenifolia*, could have arisen from A2 after the divergence of this species in the Pannonian area, where it has survived locally in lowland areas in Hungary, Bulgaria, Austria, and Moravia (Figs. 2a &2c).

Alternatively, one could also assume subclade IIa of Fig. 2a (**A2**) to be the result of hybrid introgression from *A. asplenifolia*-2x into *A. setacea*-2x. This is unlikely considering the current geographic distribution of the two diploid species and the occurrence of allele A2 among populations of *A. setacea*-2x (only in its south-eastern populations, SeAA and GR, that grow outside the distribution area of *A. asplenifolia*-2x). However, the refugia of the two species may have been in closer proximity in SE Europe during the ice-ages, and they may have hybridized there. If so, allele A2 must have been lost from *A. setacea*-2x during its northward migration. But this scenario is again unlikely because there are no signs of hybrid introgression between *A. asplenifolia*-2x and *A. setacea*-2x throughout the Pannonian area, where they often occur in close proximity. A clear separation of the two diploid species is also strongly suggested by the ncp*GS *gene tree (Fig. 1a). Thus, we assume that two *PgiC *alleles A1 and A2 existed already in the ancestral lineage and may have been sorted incompletely after the divergence of *A. asplenifolia *and *A. setacea*, while allele A3 has arisen within *A. asplenifolia *after its species separation (Fig. 2c).

In contrast to the clear genetic and morphological separation of *Achillea setacea*-2x and *A. asplenifolia*-2x, *A. collina*-4x is morphologically intermediate between these two diploid species and also linked by intermediates to other 4x-taxa of *A. millefolium *agg.. Unlike the two relic diploid species, *A. collina*-4x has widely expanded in various mesic and open vegetation types from SE and E to C Europe and is much more aggressive in disturbed habitats. From experimental crosses between *A. asplenifolia*-2x and *A. setacea*-2x, synthetic allotetraploid and *A. collina*-like plants were produced and successfully backcrossed to natural *A. collina-*4x [14]. These early results were supported by AFLP analyses which showed that species-specific bands of the two diploids are combined in *A. collina*-4x [19].

The present sequence data from single-copy nuclear genes ncp*GS *and *PgiC *(Figs. 1, 2) demonstrate that all the haplotype sequences of the diploid individuals or populations are grouped corresponding to the two species, *Achillea setacea*-2x and *A. asplenifolia*-2x respectively. In contrast, sequences of nearly all populations and many individuals of *A. collina*-4x (and its suspected 4x-hybrids) are placed among both the diploid *Achillea setacea *and *A. asplenifolia *clades. Therefore, homeologs of the nuclear single-copy genes in *A. collina*-4x demonstrate its allotetraploid origin. Additional evidence for this conclusion comes from the AFLP Neighbor Net (Fig. 3). That many of the *A. collina*-4x individuals (in the *PgiC *gene tree, most individuals) harbor homoeologous gene copies (Additional file 1 &2: Figs. S1 & S2) suggests at least partly disomic inheritance of this tetraploid species. Its diploid progenitors must have been closely related to the extant *A. setacea*-2x and *A. asplenifolia*-2x, and probably have differentiated in SE Europe. Their hybridization and the origin of an allotetraploid progeny may have taken place in the Pannonian region, where their distribution areas still overlap.

With the establishment of *A. collina*-4x, a first cycle of hybridization and differentiation was completed. But was the further expansion of this young allotetraploid species accompanied by complete isolation from or by continued backcrossing with its diploid progenitor lineages? Earlier experiments of crossing 2x- and 4x-taxa of the *A. millefolium *agg. never have produced 3x-hybrids but can occasionally gave rise to 4x-progeny via unreduced egg cells from the 2x side [14]. Such unreduced gametes occur frequently in *A. millefolium *agg. [29]. In Burgenland, Austria, populations of *A. setacea*-2x, *A. asplenifolia*-2x and *A. collina*-4x grow in two areas about 4 km apart: southeast of Rust and St. Margarethen (see Additional file 3, Table S1 for population sampling information). Ongoing gene flow may exist among their populations: Polymorphic populations M1 and M2 with mixed ploidal levels of 2x and 4x were found in disturbed grassland surrounding the morphologically more typical *A. setacea*-2x population NS1 on natural steppe islands near St. Margarethen, whilst NS1 itself also contains a few phenotypically intermediate 4x-plants. Similarly, at the outer border zone of lake Neusiedlersee near Rust, in contact zones between *A. asplenifolia*-2x in natural humid meadows and *A. collina*-4x from adjacent disturbed grassland, 4x-plants with intermediate phenotype were found in populations R1, R2 and NS2 (see Additional file 3, Table S1 for population sampling information). Our study, especially the AFLP network (Fig. 3), suggests these 4x-plants result from backcrosses of the 2x-taxa to *A. collina*-4x via unreduced female gametes. The possibility of reverse gene flow from 4x to 2x will need a further critical study.

There are several other examples for ongoing hybridization between taxa on different ploidy levels in *Achillea*: A contact zone between *A. asplenifolia*-2x and *A. collina*-4x, comparable to the one in Austria, was studied in W Hungary [30]. *A. virescens *is an allo-4x-species, which has arisen from hybridization between *A. collina*-4x and *A. nobilis*-2x. Its backcrossing with *A. collina*-4x has been demonstrated in NE Italy [18]. The yellow flowering SE-European *A. clypeolata*-2x has formed an extensive 4x-hybrid swarm with *A. collina*-4x in Bulgaria [19,31]. In addition, natural and experimental crosses between *A. collina*-4x and *A. millefolium*-6x are quite successful; via semifertile 5x-F_1_, aneuploid-F_2 _and backcrosses they rapidly produce normal euploid 4x or 6x progeny and support gene flow between the two ploidy levels [32].

## Conclusions

Combining all molecular and cytogenetic data [[11-14,19,29], etc.], we conclude that most of the polyploid taxa in *Achillea millefolium *agg. are allopolyploids or at least more or less strongly influenced by hybridization. Polyploid taxa often occur in close contact with each other and with diploids. This not only makes hybridization between polyploid taxa at the same ploidy level omnipresent, but facilitates introgression between taxa on different ploidy levels. Introgression of genetic material into diploid taxa, either from other diploid taxa or from polyploids, however, seems rare. Hybrid swarms common in natural zones of contacts between different taxa lead to the great genetic and ecological differentiation and variation of the polyploid taxa in the *A. millefolium *species complex.

## Methods

### Plant materials

For the present study, 14 populations of *A. millefolium *agg. were sampled (see Additional file 3, Table S1 for sampling information on taxa and populations): three of *Achillea asplenifolia*-2x (BZ, Ta, NS2, where NS2 contains a few individuals probably being *A. asplenifolia *x *collina*), three of *A. collina*-4x (SG, KWC, M3), four of *A. setacea*-2x (SeAA, GR, K4, NS1, where NS1 contains a few tetraploid individuals defined as *A. setacea *x *collina*), and four polymorphic "mixed" populations (R1, R2, M1, M2, where "pure" 2x-taxa occur together with suspected hybrids, forming an array of interspecific recombinations). For the AFLP analysis, the highly polymorphic populations (R1, R2, M1, M2, M3) were left out due to band complications in a trial experiment. Also excluded from the AFLP genotyping was the single-individual accession of *A. setacea *from Greece (GR). For rooting the gene trees, the uniform C-Mediterranean species *A. ligustica*-2x was used as outgroup. This is a basal species in *A*. sect. *Achillea *and sister to *A. millefolium *agg. [19,33].

Chromosome counts and DNA ploidy level determinations were conducted for the populations and individuals in this study (see Additional file 3, Table S1 for ploidy level information on each population). Young flower buds were used for chromosome counting following standard methods and DNA ploidal levels were investigated by means of propidium iodide flow cytometry [34,35] from silica gel dried leaves.

### DNA extraction

Total genomic DNA was extracted from ca. 0.02 g silica gel desiccated leaf materials following the 2x CTAB protocol [36] with slight modifications: Before the normal extraction process, sorbitol washing buffer was used to remove polysaccharides in the leaf materials (add 800 μL sorbitol buffer to the ground leaf powder → incubate the sample in ice for 10 min. → centrifuge at 10,000 g for 10 min at 4°C → add 700 μL warm 2x CTAB extraction buffer and then follow the established 2x CTAB protocol).

### PCR, cloning and sequencing of the single-copy nuclear genes

We sequenced two single-copy loci, the chloroplast-expressed glutamine synthase gene (ncp*GS*) and the cytosolic phosphoglucose isomerase gene (*PgiC*), both having a clear molecular evolutionary background and studied in other eudicots [37-45].

The ncp*GS *gene contains 12 exons and 11 introns [37]. The region from exon 7 to 11 was amplified and sequenced. Exon-primed amplifications were performed using specific primers GS-f and GS-r designed for *Achillea *(Table 1), or in some cases, amplification was first conducted with a universal primer pair GScp687f and GScp994r [40] followed by nested PCR with the *Achillea*-specific primers.

**Table 1:** Primers used for amplification and sequencing

Primer name	Primer sequence	Reference or source
GScp687f	5'-GATGCTCACTA CAAGGCTTG-3'	[40]
GScp994r	5'-AATGTG CTCTTTGTGGCGAAG-3'	[40]
GS-f	5'-AACCAATGGAGAAGTTATGC-3'	this study
GS-r	5'-CAAAACCACCTTCTTCTCTC-3'	this study
AA11F	5'-TTY GCN TTY TGG GAY TGG GT-3	[45]
Yamv (reverse)	5'-TCI ACI CCC CAI TGR TCA AAI GAR TTI AT-3'	[45]
PgiC-11F	5'-TY TGGGAYTGGGTAGGAG-3'	this study
PgiC-14F	5'-GAGTGTATGGAATGTCTC-3'	this study
PgiC-21R	5'-GGARTTGATTCCCCAAAC-3'	this study

The *PgiC *gene contains 23 exons and 22 introns [41]. The region from exon 11 to 21 was amplified and sequenced. Exon-primed amplifications were performed using *Achillea*-specific primers *PgiC*-11F and *PgiC*-21R (Table 1), or in a few cases, first with universal primers AA11F and yamv [45] and then by nested PCR using the *Achillea*-specific primers.

The amplification reaction was carried out in a volume of 20 μL containing 2 μL 10x PCR buffer, 0.5 U ex*Taq *(TaKaRa, Shiga, Japan) or HiFi (TransTaq DNA polymerase High Fidelity, TransGen Biotech), 200 μM of each dNTP, 0.2 μL DMSO, 0.5 μM of each primer, 1 μL template DNA, and ddH_2_O added to the final volume. The amplification was conducted on a Peltier thermocycler (Bio-RAD) with the following cycling scheme: 5 min at 94°C; 30 cycles of 1 min at 94°C, 30 s at 50°C, and 1.5 min at 72°C; a 15 min extension at 72°C; and a final hold at 4°C. The PCR products were electrophoresed on and excised from 1.0% agarose gel in TAE buffer. They were then purified using DNA Purification kit (TianGen Biotech or TransGen Biotech, Beijing, China). The purified PCR products were ligated into pGEM-T vector with a Promega Kit (Promega Corporation, Madison, USA). About 3-5 clones from each diploid and 5-15 from each tetraploid individual with the right insertion were randomly selected for sequencing. The plasmid was extracted with an Axyprep Kit (Axygene Biotechnology, Hangzhou, China). Cycle sequencing was conducted using ABI PRISM^® ^BigDye™ Terminator and vector primers T7/Sp6. In the case of *PgiC *gene, a third *Achillea*-specific internal primer *PgiC*-14F (Table 1) was used to sequence the entire ~1.7 kb-fragment. The sequenced products were run on an ABI PRISM™ 3700 DNA Sequencer (PE Applied Biosystems).

### AFLP genome scan

AFLP profiles were generated following established procedures [46] and PE Applied Biosystems [47]. Total genomic DNA was digested with *Mse*I and *Eco*RI. Preselective amplifications were performed using primer pairs with single nucleotides, *Mse*I-C and *Eco*RI-A, and selective amplifications using three primer combinations, *Mse*I-CAG/*Eco*RI-ACT (FAM), *Mse*I-CTT/*Eco*RI-ACC (NED) and *Mse*I-CAG/*Eco*RI-AGG (HEX). The fluorescence-labeled selective amplification products were run in a 4.5% denaturing polyacrylamide gel with the ABI Prism 377 Sequencer. Repeated restriction, amplification, and run of bands of a subset of samples (2-3 individuals per population) indicated reliability of the present AFLP data. In total, 17 individuals were used for error rate estimation [48]. Bands were scored with Genographer (version 1.6, ^©^Montana State University, 1998; http://hordeum.oscs.montana.edu/genographer/) in a size range from 50 ~ 500 bp. To avoid ambiguities, only bands with sufficient florescent intensity were scored and used as markers for analyses.

### Data analyses

Sequences were assembled with the ContigExpress program (Informax Inc. 2000, North Bethesda, MD), aligned with ClustalX 1.81, and then manually improved with BioEdit version 7.0.1. Singletons were identified via DnaSP ver. 4.10.9 [49]; they mostly could be due to PCR artefacts rather than reflect natural variability [50] and were not included in the data analyses. Majority-rule consensus sequences for clones [51] were constructed following a two-step strategy: First, the original data matrix was imported to the software DAMBE (Data Analysis in Molecular Biology and Evolution) [52] so that multiple sequences belonging to the same haplotype were combined into one, and the thus retained data set was used for an initial phylogenetic analyses; second, following the initial phylogenetic analysis, the number of sequences was further reduced by eliminating some suspected PCR-recombinant sequences (see Additional file 4) and by combining several polytomic haplotypes into one. Such retained data set of consensus sequences was used for the final phylogenetic analyses. These consensus sequences are labeled by the population codes and the number (amount) of individuals and clones (Figs. 1 &2). Those used as consensus sequences were deposited in the NCBI GenBank under accession numbers FJ434254-FJ434336.

Phylogenetic analyses were performed separately on the *PgiC *and the ncp*GS *data sets with PAUP* version 4.0b10a using both Maximum Parsimony (MP) and Neighbor Joining (NJ) methods. All nucleotide substitutions were equally weighted. Gaps were treated as missing data. For the MP method, heuristic searches were performed using 1000 random taxon addition replicates with ACCTRAN optimization and TBR branch swapping. Up to 10 trees with scores larger than 10 were saved per replicate. The stability of internal nodes of the MP tree was assessed by bootstrapping with 1000 replicates (MulTrees option in effect, TBR branch swapping and simple sequence addition). The NJ analysis was conducted with Kimura's 2-parameter distances [53] and bootstrapped with 1000 replicates.

Earlier reconstruction of the phylogeny of *Achillea millefolium *agg. using AFLP data showed that only the relationships of the diploid taxa conform to a bifurcating tree. Inclusion of the polyploid taxa, however, destabilizes the tree to such an extent that the distinctness of related groups becomes blurred [11,19]. Phylogenetic networks should be preferred over phylogenetic trees when reticulate events are to be expected as is the case here [54]. Therefore, the present AFLP data were analyzed using the Neighbor-Net method [55] with uncorrected p-distances embedded in SplitsTree4. In the network, parallel edges represent splits of taxa/populations, while nodes that connect incompatible splits often represent taxa/populations with hybrid origin (though conflicting signals could also be caused by homoplasy or methodological artifacts) [54].

## Authors' contributions

JXM and YNL performed the lab work, participated in the data analysis and helped to draft the manuscript. CV participated in the design of the study, collected part of the plant samples and provided input on manuscript drafting. YPG and FE conceived the project and collected most of the plant samples. FE identified all plant materials and provided significant input on manuscript drafting, whereas YPG conducted the final statistical analysis and drafted the manuscript. All authors read and approved the final manuscript.

## Supplementary Material

Additional file 1 **Fig. S1 The 50% majority-rule consensus MP tree corresponding to Fig. **1b** with original labels of the terminal nodes**. In Fig. S1, we provide original labels for terminal nodes which are simplified in Fig. 1. Click here for file

Additional file 2 **Fig. S2 The 50% majority-rule consensus MP tree corresponding to Fig. **2b** with original labels of the terminal nodes**. In Fig. S2, we provide original labels for terminal nodes which are simplified in Fig. 2. Click here for file

Additional file 3 **Table S1 Taxa and populations studied**. In Table S1, we provide the sampling information on taxa and populations, e.g., their names, geographic localities and habitats, ploidy levels as well as number of individuals and cloned sequences analyzed by this study. Click here for file

Additional file 4 **A list of sequences obtained by this study and those deleted for the final data analyses**. In this list, we highlight the sequences deleted during our final data analyses. These sequences might contain PCR artefacts, e.g., PCR-mediated recombination which is inevitable when sequencing nuclear genes from genomes where two partially homologous templates exist. We further briefly discuss the methods to avoid such artefact in experiments and to identify PCR-recombinant sequences in data analysis. Click here for file
