# Supplementary figures and images for: Allopolyploid speciation and ongoing backcrossing between diploid progenitor and tetraploid progeny lineages in the Achillea millefolium species complex: analyses of single-copy nuclear genes and genomic AFLP
Source: BMC Evol Biol. 2010 Apr 13;10:100. doi: 10.1186/1471-2148-10-100 (PMC2873412; doi:10.1186/1471-2148-10-100)

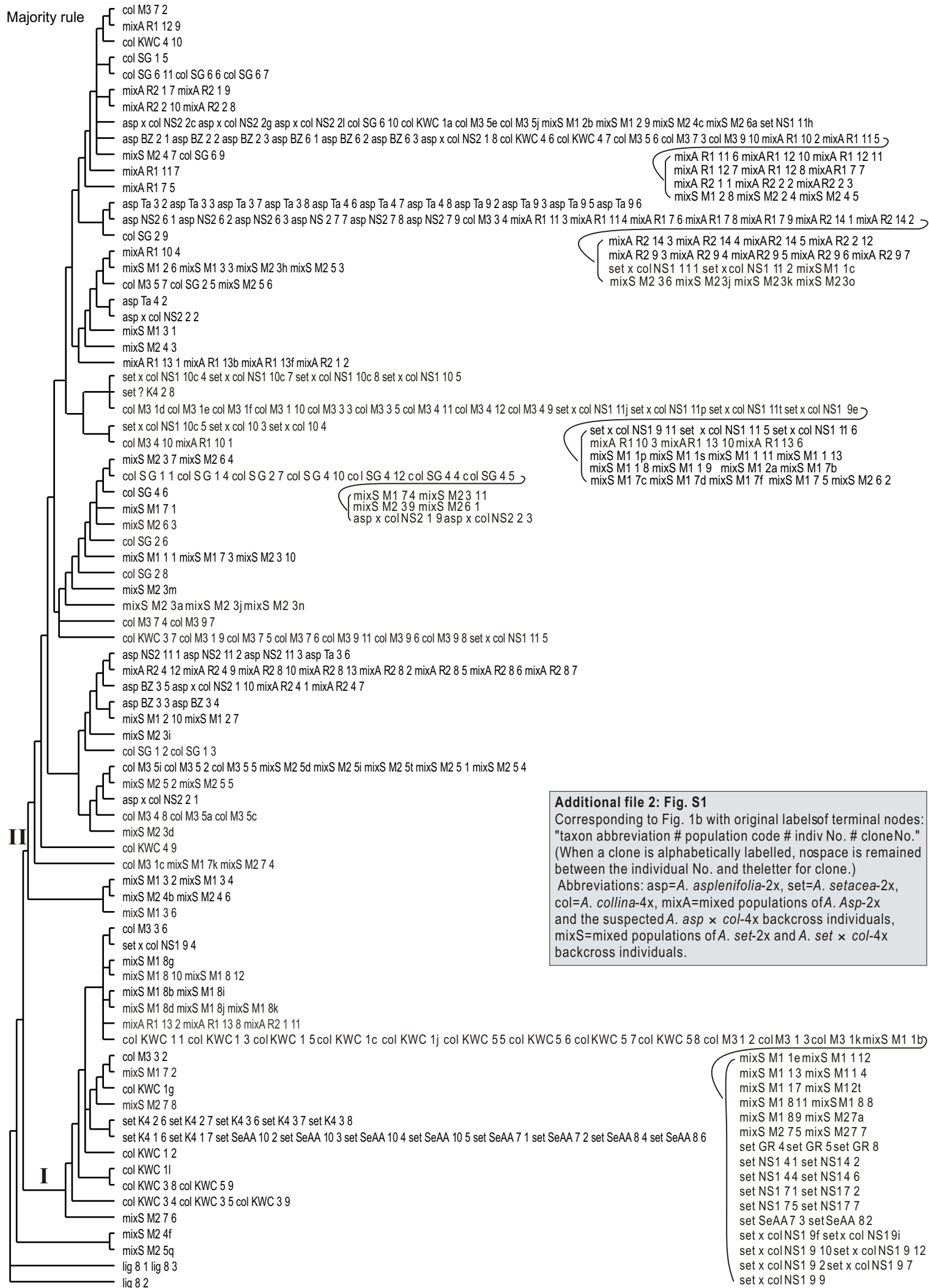

Supplement: Additional file 1 — Fig. S1 The 50% majority-rule consensus MP tree corresponding to Fig. 1b with original labels of the terminal nodes. In Fig. S1, we provide original labels for terminal nodes which are simplified in Fig. 1. [file 1471-2148-10-100-S1.PDF]
