# Supplementary material for: Allopolyploid speciation and ongoing backcrossing between diploid progenitor and tetraploid progeny lineages in the Achillea millefolium species complex: analyses of single-copy nuclear genes and genomic AFLP
Source: BMC Evol Biol. 2010 Apr 13;10:100. doi: 10.1186/1471-2148-10-100 (PMC2873412; doi:10.1186/1471-2148-10-100)
Supplement: Additional file 3 — Table S1 Taxa and populations studied. In Table S1, we provide the sampling information on taxa and populations, e.g., their names, geographic localities and habitats, ploidy levels as well as number of individuals and cloned sequences analyzed by this study. [file 1471-2148-10-100-S3.DOC]

J-X Ma, Y-N Li, C Vogl, F Ehrendorfer and Y-P Guo: Allopolyploid speciation and ongoing backcrossing between diploid progenitor and tetraploid progeny lineages in the *Achillea millefolium* species complex: analyses of single-copy nuclear genes and genomic AFLP

**Additional file 1:**

**Table S1** Taxa and populations studied

| Taxa | Pop. code | Ploidy level | Locality and habitat | Collecting information^1^ | No. indiv. / clones studied^2, 3^ | | | | |
| --- | --- | --- | --- | --- | --- | --- | --- | --- | --- |
|  |  |  |  |  | Chrom. counts | Flow cyto-metry | ncp*GS* | *PgiC* | AFLP |
| *A. asplenifolia* Vent. | BZ | 2x | Austria: Burgenland, between Weiden and Podersdorf; ca. 120 m, natural humid meadows | Tod, 2001.10.03 | 2 | 1 | 3 / 9 | 3 / 12 | 13 |
|  | Ta | 2x | Czech Rep.: Southern Moravia, Terezin; ca. 200 m, natural humid meadows | FE & LE, 2002.07.12 |  | 3 | 3 / 13 | 3 / 8 | 13 |
|  | NS2 (with 2 marginal individuals, NS2_1 & 2, defined as *A. asplenifolia* × *collina*) | 2x  +  4x | Austria: Burgenland, SE of Rust; ca. 115 m, natural humid meadows towards lake Neusiedler See | FE, JS & YPG, 2003.05.27 | 2  +  2 |  | 3 / 9  +  2 / 9 | 3 / 12  +  2 / 10 | 11 |
| *A. collina* J. Becker ex Heimerl | SG | 4x | Austria: Carinthia, St. Veit / Glan, Längsee; ca. 580 m, dry grassland | FE, 2001.07.22 |  | 4 | 4 / 20 | 3 / 15 | 9 |
|  | KWC | 4x | Austria: Lower Austria,Kaltenleutgeben, Wiener Hütte; ca. 400 m, dry grassland | YPG, 2002.06.27 |  | 3 | 4 / 23 | 4 / 19 | 12 |
|  | M3 | 4x | Austria: Burgenland, 2.5 km E of St. Margarethen; ca. 200 m, dry grassland distant from *Achillea setacea* | FE & CV, 2006.07.05 | 3 | 2 | 6 / 38 | 6 / 28 |  |
| *A. setacea* Waldst. & Kit. | GR | 2x | Greece: NE Thessaloniki, drain from lake Limni Koronia; ca.100 m | JS (FE, 2001) |  | 1 | 1 / 3 | 1 / 3 |  |
| *A. setacea* Waldst. & Kit. | SeAA | 2x | Turkey: Anatolia, Aksaray; ca. 950 m, dry grassy and ruderal places | FE, 2002.03.26 | 1 | 2 | 3 / 10 | 3 / 10 | 10 |
|  | K4 (one individual is probably polyploid) | 2x | Ukraine: Kiev, Bald Mt.= Lisa Gora, S of the City; ca.130 m, dry grassland | FE & YPG, 2003.07.22 | 2 | 1 | 3 / 8 | 3 / 9 | 11 |
|  | NS1 (with 5 marginal 4x-dividuals, NS1_9, 10, 10c & 11 defined as *A. setacea* × *collina* | 2x  +  4x | Austria: Burgenland, 2.5 km E of St. Margarethen; ca. 200 m, natural steppes on limestone | FE, JS & YPG, 2003.05.27 | 2  +  3 |  | 2 / 8  +  4 / 26 | 2 / 6  +  4 / 17 | 9  +  5 |
| *A. asplenifolia +*  *A. asplenifolia* × *collina*  (defined as mixA) | R1 | 2x+4x | Austria: Burgenland: SE of Rust; ca. 115 m; agriculturally disturbed vegetation towards lake Neusiedler See | FE & CV, 2006.07.05 | 3 | 2 | 5 / 26 | 5 / 24 |  |
|  | R2 | 2x+4x | adjacent to the foregoing | FE & CV, 2006.07.05 | 2 | 4 | 6 / 30 | 6 / 32 |  |
| *A. setacea* *+*  *A. setacea* × *collina*  (defined as mixS) | M1 | 2x+4x | Austria: Bugenland, 2.5 km E of St. Margarethen; ca. 200 m, disturbed grassland between “pure” *Achillea setacea* and *A. collina*, limestone | FE & CV, 2006.07.05 | 2 | 2 | 5 / 48 | 5 / 23 |  |
|  | M2 | 2x+4x | adjacent to the foregoing | FE & CV, 2006.07.05 |  | 3 | 5 / 44 | 5 / 21 |  |
| *A. ligustica* | SN | 2x | Italy: Sicily, Nebrodi Mts. | FE, 2001.09.21 |  | 1 | 1 /3 | 1 / 3 |  |

**Notes:**

^1^ Names of collectors: CV = C. Vogl; FE = F. Ehrendorfer; JS = J. Saukel, LE = L. Ehrendorfer-Schratt; YPG = Y.-P. Guo.

^2^ The same 60 individuals were sequenced for both gene loci except one, *A. collina*_SG_2, which was eliminated from the *PgiC* data set due to the continuous failure to obtain the right fragment from this individual. All these individuals were also included in the AFLP analysis. Vouchers are deposited in the herbaria of the Institute of Botany (WU) and the Institute of Pharmacogonosy, both at the University of Vienna, Austria.

^3^ NCBI GenBank accession numbers for sequences from this study are FJ434254–FJ434336.
